# Depression in patients with chronic pain attending a specialised pain treatment centre: prevalence and impact on health care costs

**DOI:** 10.1097/j.pain.0000000000000542

**Published:** 2016-03-08

**Authors:** Lauren Rayner, Matthew Hotopf, Hristina Petkova, Faith Matcham, Anna Simpson, Lance M. McCracken

**Affiliations:** Departments of a Psychological Medicine and; b Health Services and Population Research, Institute of Psychiatry, Psychology and Neuroscience, King's College London, London, United Kingdom; c Health Psychology Section, Institute of Psychiatry, Psychology and Neuroscience, King's College London, London, United Kingdom; d INPUT Pain Management Centre, Guy's and St Thomas' NHS Foundation Trust, London, United Kingdom

**Keywords:** Depression, Chronic pain, Health care utilization, Health care costs

## Abstract

This cross-sectional study aimed to determine the prevalence and impact of depression on health care costs in patients with complex chronic pain. The sample included 1204 patients attending a tertiary pain management service for people with chronic disabling pain, unresponsive to medical treatment. As part of routine care, patients completed a web-based questionnaire assessing mental and physical health, functioning, and service use in the preceding 3 months. Depression was assessed using the 9-item Patient Health Questionnaire. Self-report health care utilisation was measured across 4 domains: general practitioner contacts, contacts with secondary/tertiary care doctors, accident and emergency department visits, and days hospitalised. The participation rate was 89%. Seven hundred and thirty-two patients (60.8%; 95% CI 58.0-63.6) met criteria for probable depression, and 407 (33.8%) met the threshold for severe depression. Patients with depression were more likely to be unable to work because of ill health and reported greater work absence, greater pain-related interference with functioning, lower pain acceptance, and more generalised pain. Mean total health care costs per 3-month period were £731 (95% CI £646-£817) for patients with depression, compared with £448 (95% CI £366-£530) for patients without depression. A positive association between severe depression and total health care costs persisted after controlling for key demographic, functional, and clinical covariates using multiple linear regression models. These findings reveal the extent, severity, and impact of depression in patients with chronic pain and make evident a need for action. Effective treatment of depression may improve patient health and functioning and reduce the burden of chronic pain on health care services.

## Abstract 2

Depression in chronic pain is highly prevalent (60.8%) and is a significant predictor of poorer functioning, pain interference, reduced work status, and increased health care costs.

## 1. Background

Chronic pain and depression are each prevalent and often co-occur.^5^ Although the underlying mechanism of the interaction between pain and depression is not fully understood, their coexistence has been shown to incur additive adverse effects on patient outcomes, including poorer functioning and reduced response to treatment.^2,36^ The systematic review of Bair et al.^5^ revealed wide variation in estimates of the prevalence of depression in patients with chronic pain. Estimates ranged from 4.7% to 22% in population-based studies and from 5.9% to 46% in primary care studies. Variability in estimates was particularly striking in studies of specialist pain populations (1.5%-100%). Most of these studies, however, were conducted in the 1980s and included fewer than 100 participants. More recent studies of depression in specialist pain settings have yielded prevalence estimates ranging from 12.1% to 72%, but sample sizes remain small.^20,27,28,33^

Chronic pain poses an enormous economic burden on society both in terms of medical care and lost productivity. A National Institutes of Health report in 2011 estimated the total annual incremental cost of health care due to pain in the United States at $261-$300 billion. The total cost to society, including indirect costs of pain due to lower economic productivity, was estimated at $560-$635 billion—greater than the cost of heart disease, cancer, or diabetes.^21^ Given the magnitude of these costs, surprisingly, few studies have examined the factors associated with health care utilisation in people with chronic pain. Blyth et al.^8^ found that a high level of pain-related interference was associated with a greater number of general practitioner (GP) and emergency department visits and more frequent hospitalisation, in the general population. Azevedo et al.^4^ identified depressed mood and pain-related disability as the main drivers of increased medical consultations, and Andersson et al.^1^ found that people with chronic pain and depressive symptoms made more physician visits than those with pain alone. These population-based studies indicate that pain interference and depression may predict health care use, but there remains a dearth of data from more complex, multiproblem, specialist pain populations.

If depression modifies the relationship between chronic pain and health care utilisation, there may be opportunities to reduce the burden of chronic pain on both the individual and society, through improved detection and treatment of the disorder in this context. Up to date evidence on the prevalence and impact of depression in specialist pain services is therefore necessary to determine the need for increased investment in mental health resources for this population. The aims of this study were (1) to determine the prevalence of depression in a specialist pain population and (2) to describe the impact of depression on health care utilisation and costs.

## 2. Methods

### 2.1. Participants

The study sampling frame was all patients attending an initial assessment consultation at the INPUT pain unit at Guy's and St Thomas' NHS Foundation Trust in central London, United Kingdom, between February 2013 and June 2015. The INPUT pain unit is a specialty interdisciplinary pain management service that provides residential, outpatient, and individual treatment for patients with chronic pain. Generally, the referral population for the service includes people with chronic pain (typically greater than 9 months), and disability, who have sought previous primary care and hospital care, such as pain clinic, orthopedics, or rheumatology services, for their pain without satisfactory resolution. Most referrals are from the south east of England. The INPUT pain unit is a typical U.K. tertiary care service.

### 2.2. Procedure

Data were collected as part of routine care using the IMPARTS (Integrating Mental and Physical healthcare: Research Training and Services) web-based screening interface.^29^ IMPARTS is a multifaceted service development package designed to improve integration of mental and physical health care. It does this through (1) routine measurement of mental and physical patient-reported outcomes, with real-time feedback and decision support through the electronic patient record; (2) development of mental health referral pathways for individuals with identified mental health needs; (3) training staff in core mental health skills; and (4) development of self-help materials specific to the condition in question. Further information about IMPARTS has been reported elsewhere.^29^ The INPUT pain unit implemented IMPARTS in February 2013 to facilitate systematic assessment of depression alongside other outcomes pertinent to the chronic pain population. Patients were approached to participate in screening at their first visit to INPUT, which is an assessment consultation to determine their suitability for the treatments provided. Consecutive patients attending the assessment clinic were invited by reception staff to complete a questionnaire on a touchscreen e-tablet in the waiting room, before their consultation. IMPARTS establishes routine outcome measurement as a service development rather than as a research project. Therefore, formal consent was not required, but patients were informed and given information sheet explaining that completion of the questionnaire was voluntary and that responses would be confidential and only used for audit or research if fully anonymised. On completion of the questionnaire, patients' screening results immediately populated their electronic patient record, ready for the clinical team to review.

IMPARTS has research ethics approval from the National Research Ethics Service Research Database Committee (NRES, Ref: 12/SC/0422), which permits the use of de-identified data collected through IMPARTS for research purposes, with the added safeguard that each project was approved by a patient-led research oversight committee.

### 2.3. Clinical outcome measures

Measures were selected for inclusion in the IMPARTS questionnaire in collaboration with the INPUT clinical team, based on appraisal of the existing scientific literature. The questionnaire was necessarily brief so that it could be completed in the course of routine care, with minimal impact on staff and patients' time.

Depression was assessed using the 9-item Patient Health Questionnaire (PHQ-9),^35^ which has been validated in patients with a broad range of physical health problems, including chronic pain.^9^ A meta-analysis of 113 studies examining the diagnostic accuracy of depression case-finding tools in chronic illness showed that the PHQ-9 had high sensitivity (0.84; CI 0.69-0.91) and specificity (0.88; CI 0.83-0.91) compared with other commonly used screening tools such as the Hospital Anxiety and Depression Scale and the General Health Questionnaire 12.^25^ Choi et al.^9^ found that the PHQ-9 was among the best-performing tools (area under the curve, 0.796 [SE, 0.022]) in their comparison of case-finding instruments for depression in patients with chronic spinal pain.

Criteria for probable major depressive disorder (MDD) were met if the patient reported low mood or loss of interest and at least 5 of 9 symptoms in total, for more than half the days in the last 2 weeks. Item 9 (suicidal thoughts) counted towards the diagnosis of MDD if present at all. Severe depression was defined as MDD caseness with PHQ-9 score 20 to 27, moderate depression was defined as MDD caseness with PHQ-9 score 15 to 19, and mild depression was defined as MDD caseness with PHQ-9 score <15. Suicidal ideation was assessed by item 9 of the PHQ-9 and defined as having “thoughts that you would be better off dead or of hurting yourself in some way” for more than half the days in the previous 2 weeks. Pain acceptance was measured using the 8-item version of the Chronic Pain Acceptance Questionnaire (CPAQ).^24^ The CPAQ-8 measures the extent to which the patient is able to experience pain without struggling to control or avoid it. It has a 2-factor structure: pain willingness (disengagement from efforts to control or avoid pain) and activity engagement (doing valued activities despite the presence of pain). Level of pain acceptance is measured on a 0 to 6 rating scale. Pain willingness, activity engagement, and total pain acceptance scores are calculated by summing the responses in each domain. Psychometric evaluations of the short-form CPAQ-8 have shown the same factor structure as the original 20-item version, with good reliability and validity.^15,31^ Low pain acceptance has been found to be strongly associated with depression, independent of pain intensity.^22,38^

### 2.4. Resource use and cost

Self-report resource use data were collected as part of the IMPARTS questionnaire. Four domains of health care service use for pain in the preceding 3 months were measured: (1) GP contacts, (2) contacts with other doctors (eg, secondary/tertiary care), (3) accident and emergency department (A&E) visits, and (4) days hospitalised. For example, patients were asked “How many times have you seen your GP in the past 3 months for your pain?” To monetarily value patient-reported health care utilisation, each service was assigned a unit cost. Health care costs were calculated by multiplying each resource use item by the unit cost. All unit costs, in British Pound Sterling, were from the financial year 2013 to 2014 and were obtained from sources detailed in Table 1.

**Table 1.**
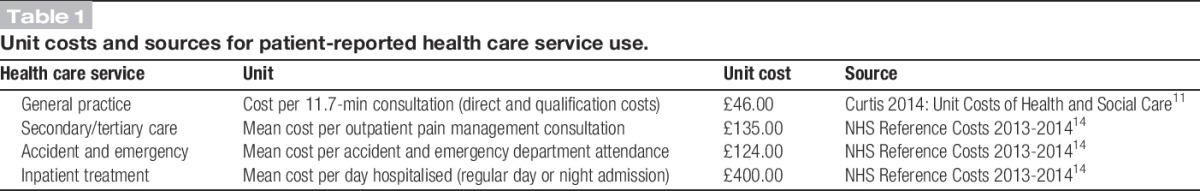
Unit costs and sources for patient-reported health care service use.

Pain interference is associated with depression and has been shown to predict health care utilisation in population-based studies.^5^ We assessed pain interference using the Brief Pain Inventory (BPI).^13^ The pain interference dimension of the BPI comprises 7 items measuring the degree to which pain interferes with functioning in the following domains: general activity, mood, walking ability, normal work, relations with other persons, sleep, and enjoyment of life. Level of interference is measured using a rating scale from 0 (no interference) to 10 (complete interference), and the mean score denotes the total level of pain interference. The BPI has been widely used and validated as a measure of pain interference in daily functioning.^37^ Research indicates that pain interference is a powerful independent predictor of depression^12^ and may also be a driver of higher health service costs.^8^

Pain relief provided from treatments or medications was also measured by the BPI. Question 7 asks the patient to select the percentage that best indicates the extent of relief, on a 10-point scale from 0% to 100%.^13^ Additionally, patients were asked about the duration, location, and extent of their pain and whether they experienced generalised pain (yes/no). Smoking status and occupational functioning were also assessed.

### 2.5. Data analysis

Prevalence and severity of probable depression were expressed as the percentage of cases determined by the PHQ-9, with 95% CI. The sociodemographic and clinical characteristics of patients with and without depression were compared using the χ^2^ test for categorical data and the Mann–Whitney *U* test for continuous data that were not normally distributed. For ordinal data, test for trend was calculated using logistic regression, with *P* values derived from the Wald test. Mean costs were compared using the Student *t*-test. Inferences about average cost differences should always be based on a comparison of the arithmetic mean, even though cost data are often skewed.^6^

Multivariate analysis of the impact of depression on health care costs was conducted using multiple linear regression models (ordinary least squares). We expected health care costs to vary by depression severity; therefore, for the regression analysis, we categorised depression into 4 categories (none/mild/moderate/severe). The purpose of the regression analysis was not to find the best model to explain variability in health care costs but rather to test the hypothesis that depression is predictive of cost whilst controlling for potential confounders. Potential adjustment variables were selected based on established associations identified in the literature and statistical relationships with outcome and exposure variables detected in the data. Automated selection procedures and model fitting based solely on significance testing may result in inappropriate inclusions and exclusions. Although there is no agreed optimal strategy for modelling, confounder identification should be based on a priori knowledge about associations and causal pathways (research evidence and expert judgement) in addition to statistical information.^18^ Given that there is uncertainty in the literature regarding the causal network linking depression, health care costs, and potential confounders such as pain severity, we computed 4 models with varying levels of adjustment, to examine how selection of adjustment variables impacted the relationship between depression and health care costs: model 1, unadjusted; model 2, adjusted for age and sex; model 3, additionally adjusted for occupational status; model 4, additionally adjusted for pain interference, pain acceptance, and generalised pain. Health care costs were skewed to the right; therefore, nonparametric bootstrapping (1000 replications) was used to estimate bias-corrected and accelerated confidence intervals around cost differences. All statistical analyses were performed using Stata 11 software.

## 3. Results

During the study period (February 2013 to June 2015), 1352 patients attended an assessment consultation at the INPUT pain unit, and of these, 1204 (89%) completed the IMPARTS questionnaire. Data on the reasons for nonparticipation were not recorded, but clinic staff reported that the most common reasons were patients arriving late and an inadequate number of e-tablets being available.

### 3.1. Sample characteristics

Two-thirds of the sample were women, and the median age was 47 (interquartile range, 38-53). Just over half of the sample (52.8%) reported being unable to work for health reasons, whereas 22% was currently employed. The median duration of pain was 7 years. Lower back pain was the most frequent primary complaint, and 88% of the sample reported experiencing generalised pain (Table 2).

**Table 2.**
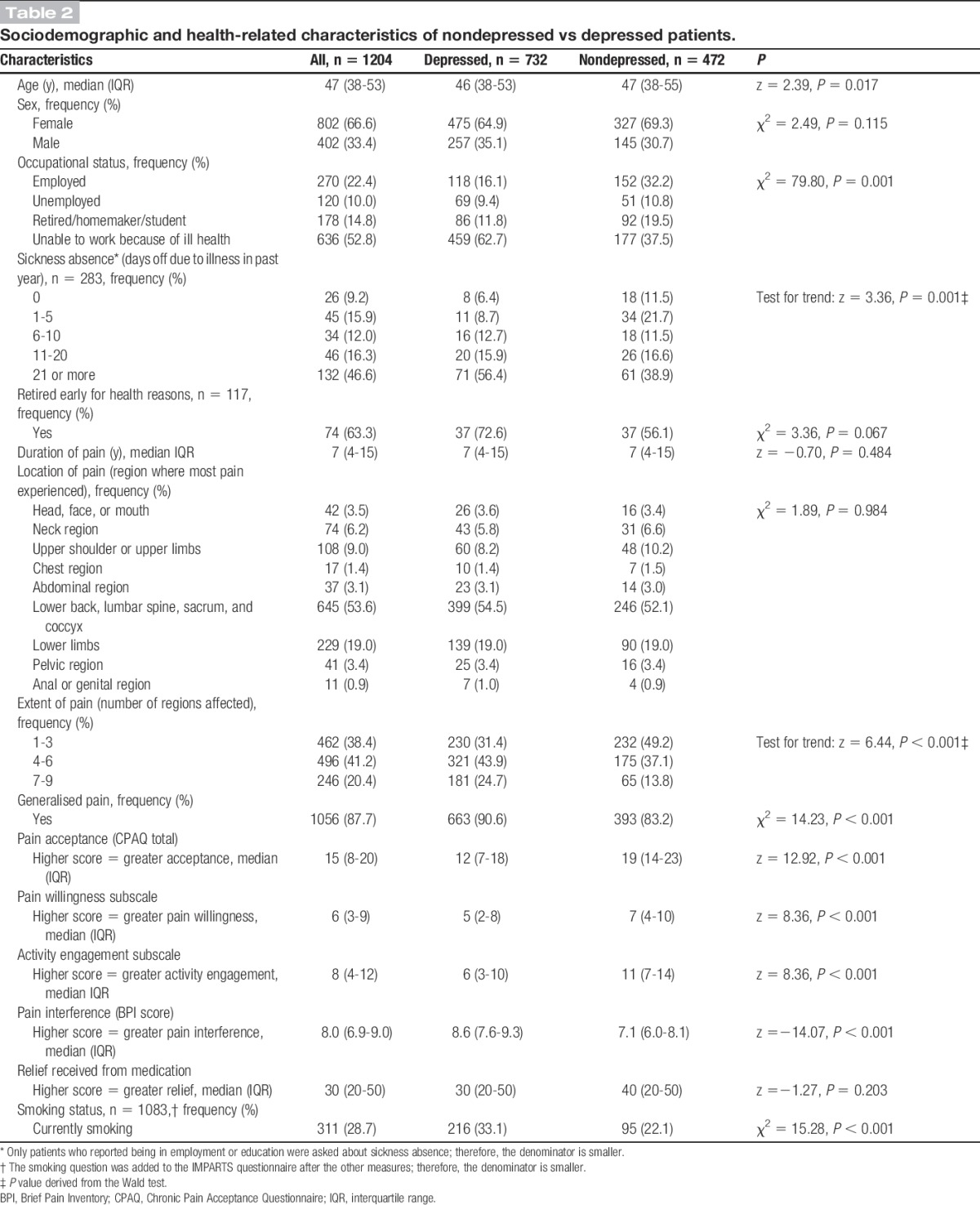
Sociodemographic and health-related characteristics of nondepressed vs depressed patients.

### 3.2. Prevalence of depression

According to the PHQ-9, 732/1204 patients (60.8%; CI 58.0-63.6) met criteria for probable MDD. Sixty-nine patients (5.7%) met the threshold for mild MDD, 256 (21.3%) met the threshold for moderate MDD, and 407 (33.8%) met the threshold for severe MDD. One hundred eighty-three of 1204 patients (15.2%) reported suicidal thoughts.

### 3.3. Demographic and clinical characteristics of depressed vs nondepressed patients

Table 2 compares the demographic and clinical characteristics of chronic pain patients with and without depression. There was no difference in gender, but a small statistically significant difference in median age between depressed and nondepressed patients (46 vs 47). More patients with depression reported being unable to work because of ill health, and of those in employment, depressed patients were more likely to have taken >21 days of sickness absence in the past year. The extent of pain (number of regions affected) was greater for patients with depression. Patients with depression were also more likely to report having generalised pain. Depressed patients had reduced pain acceptance, scoring lower overall on the CPAQ-8, and also on the pain willingness and activity engagement subscales. Patients with depression experienced greater pain interference that those without depression and were also more likely to smoke. Depressed and nondepressed patients did not differ regarding the duration of pain or the extent of relief received from medication.

### 3.4. Descriptive analyses: depression and health care costs

Table 3 compares resource use and costs per 3 months for patients with and without depression. Compared with those without depression, a larger proportion of depressed patients visited a GP for their pain in the 3 months preceding IMPARTS assessment (87% vs 77%). Depressed patients were also more likely to have contact with other doctors (71% vs 67%), attend A&E (18% vs 13%), and be admitted to hospital (13% vs 7%). Mean costs per patient were also higher for patients with depression than for those patients without depression. A statistically significant cost difference was observed in every health care domain, but the difference was greatest for hospital admissions, where expenditure for depressed patients was more than twice that for nondepressed patients (£266; CI £147-£384 vs £101; CI £38-£164). Total health care costs per participant over 3 months were estimated at £731 (CI £603-£861) for patients with depression, compared with £448 (CI £366-530) for patients without depression (*P* = 0.001).

**Table 3.**
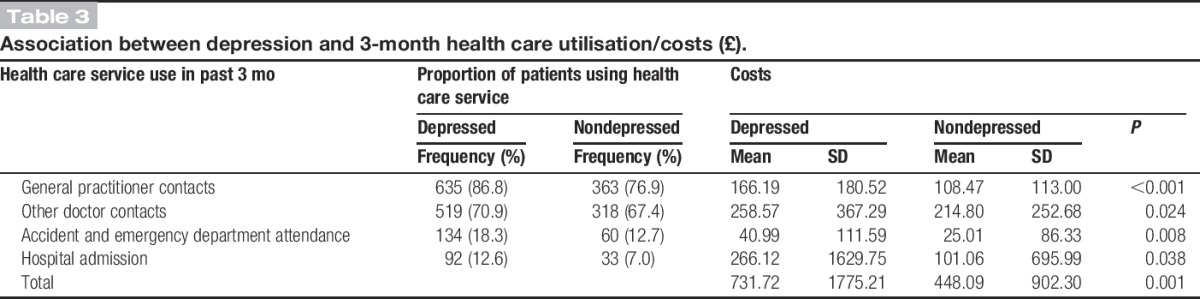
Association between depression and 3-month health care utilisation/costs (£).

### 3.5. Multivariate analysis

Table 4 presents the results of the multiple regression analyses by health care domain, with 3-month costs as the dependent variable. It shows that moderate and severe depression had a significant impact on total health care costs per 3 months, which was attenuated but not eliminated with adjustment for potential confounders. In model 2, adjusted for age and gender, the relationship between depression and health care costs remained virtually unaltered, with only a marginal decrease in mean cost differences across health care domains. In model 3, additional adjustment for occupational status resulted in further, but again modest, reductions in mean cost differences. The association between moderate/severe depression and health care costs persisted with the addition of pain variables (generalised pain, pain acceptance, and pain interference) in model 4, although a more pronounced attenuation of the effect was observed, particularly for “hospital admission” and “other doctor” costs. Severe depression increased 3-month total health care costs by £248 (CI £43-£507). Moderate depression increased total health care costs by £134 (CI £-52-£364), although the effect did not reach statistical significance. No effect was observed for mild depression. Among domain-specific costs, depression had a significant impact on GP costs (*P* < 0.001), with moderate and severe depression increasing 3-month costs by £48 (CI £21-£78) and £43 (CI £20-£66), respectively. Severe depression also increased costs for A&E attendance, hospital admission, and other doctor contacts, although these effects were not statistically significant.

**Table 4.**
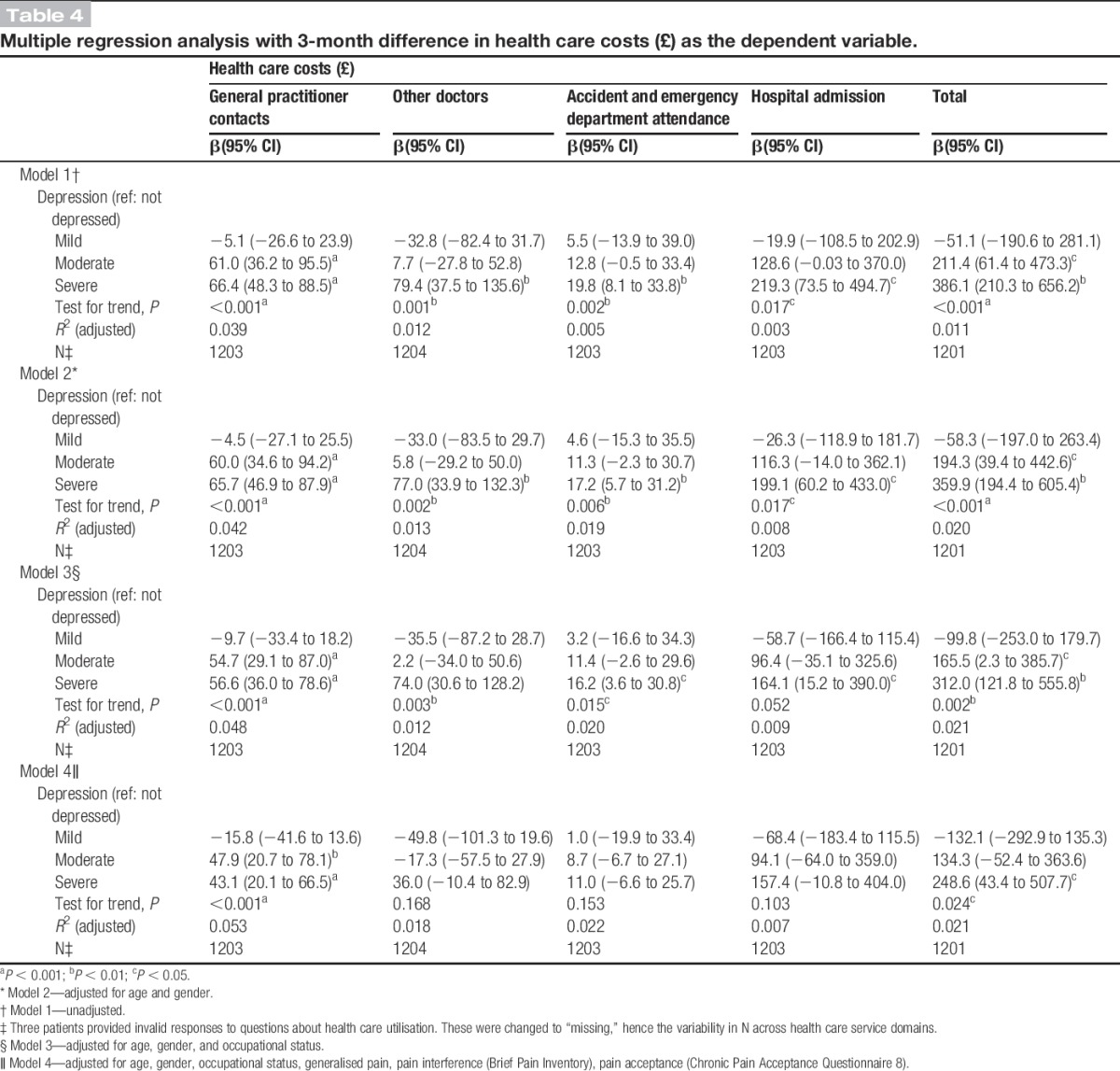
Multiple regression analysis with 3-month difference in health care costs (£) as the dependent variable.

## 4. Discussion

This study documents a high prevalence of depression in people with chronic pain, 60.8%, based on a standard self-report measure in a large specialty care sample. The symptoms of depression reported in this group reflected a high severity, with 55.6% of those meeting criteria reporting a severe level of symptoms or 33.8% of the total sample. Overall, patients who met criteria for depression were more likely not to work because of ill health and reported greater work absence, more generalized pain, greater pain-related interference with functioning, lower pain acceptance, and greater health care use and costs, relative to those who did not meet these criteria. These patients were also more likely to smoke.

Depression was linked to greater health care use in each of the services assessed, including GP visits, other doctor visits, A&E visits, and hospital admissions. The greater use of A&E and hospital admission was particularly notable, proportionally 31% and 44% greater, respectively. In multivariate analyses, which included key covariates, the association between depression and higher health service costs persisted for patients with more severe symptomatology. In the fully adjusted model, we found significantly higher total health care costs for patients with severe depression. General practitioner costs also remained significantly higher for patients with moderate-to-severe depression. The persistence of the effect indicates that the association between depression and health care costs is independent of pain severity.

The range of available prevalence estimates suggests that depression is a substantial problem among patients with chronic pain. This study updates and underscores this finding. The prevalence of depression observed here is somewhat higher than the average for other recent studies of this same type of population, those seeking specialty treatment for chronic pain.^20,27,28,33^ Methodological differences—most notably the use of different case-finding tools—may have led to the apparent variation or there may be another explanation, such as a local factor relating to the region or service where this study was conducted. For example, the INPUT pain unit is a tertiary care service. Patients referred here have previously sought primary and secondary care for their pain without satisfactory resolution and thus may be at increased risk of depression. Preferential referral of patients presenting with psychological distress may also partially account for the high prevalence of depression in this patient group. Our single centre design limits the extent to which we can make general statements regarding the study findings. Health care utilisation and depression might be higher in this sample than in other pain populations because both may predict entry to the INPUT unit. Even those classified as nondepressed may have had relatively high depressive symptomatology, resulting in a smaller association between depression and health care costs than would be observed in populations with greater variability. Further study is needed to test the reliability of our results and their applicability to different settings.

A notable limitation of this study is the potential for misclassification of exposure. The PHQ-9 is a self-report screening tool that has been shown to have high sensitivity and specificity compared with other case-finding tools.^25^ It nonetheless lacks the depth and diagnostic validity of the gold standard clinical interview and will both miss cases and identify cases erroneously. The PHQ-9 uses the 9 core DSM-V criteria for depression but does not take account of exclusionary criteria that should preclude diagnosis—ie, that symptoms should not be caused by substance misuse or a general medical condition nor be better accounted for by bereavement. Furthermore, several symptoms assessed by the PHQ-9, such as difficulty concentrating and sleep disturbance,^3^ may be attributable to depression or pain. It is therefore possible that some study participants reporting these symptoms were misclassified as depressed, resulting in an inflated estimate of depression prevalence and dilution of the true strength of the association between depression and health care costs. Additionally, it should be noted that temporally, health care utilisation preceded measurement of the PHQ-9. In assessing depression subsequent to health care spending, our analysis assumes that depression is a relatively stable behaviour pattern that also preceded the health care. Yet, it is possible that some patients who screened positive for depression on the PHQ-9 were not depressed in the previous 3 months, and some who screened negative may have only recently remitted. Nevertheless, the impact of depression instability on our results is likely to be small,^26^ and we believe it is plausible that depressed patients with high health care costs in the preceding 3 months would continue to have high costs in subsequent months.

The possibility of inaccurate recall of health care utilisation is another limitation. In this analysis, health care utilisation, and therefore health care costs, relied on information collected from patients. Self-report is an efficient, widely used method of assessing service use, recognised for its simplicity and cost efficiency compared with retrieval of medical records.^34^ These attributes make self-report particularly suitable for studies with large samples and those assessing several domains of health service use. Studies in general medical and mental health populations have shown good levels of reliability and high congruency rates between self-report and medical records for recall periods of up to 12 months.^7,17^ Nevertheless, the most accurate measure of service use is obtained by using multiple sources, and our reliance on a single method of data collection is a limitation that we acknowledge. Furthermore, Rozario et al.^32^ found that depression was a marginally significant factor in predicting lower congruence between self-report and medical record data, and there is some evidence that severely depressed patients may overreport health care utilisation.^30^ It is therefore possible that recall bias may have inflated the association between severe depression and health care costs observed in this study.

A limited number of variables were assessed in this study, necessarily so, as the routine assessment process needed to be short. Potentially, there are other confounding variables that, if available, could have led to differing conclusions about associated factors, impacts, service use, and costs. For example, illness perceptions have been shown to be associated with both depression^10^ and health care utilisation, with illness worry, a long timeline perspective, and belief in serious consequences, predicting high service use.^16^ Differences in underlying comorbidity and ongoing treatment of depression are also possible confounders. Patients with chronic conditions requiring regular medical intervention and those being treated for depression are likely to see their GP more frequently. Although patients were asked to report specifically on health service use for pain, some may have found it difficult to disentangle the primary reason for their consultation, and this may have led to an overestimation of the association between depression and GP visits in our sample. It would also have been helpful to include sociodemographic information such as ethnicity and social deprivation in the analysis. However, a high level of data errors and omissions from clinical records meant that these data were not consistently accurate or available.

We adopted a narrow costing approach, focusing on the impact of comorbid depression on health care service costs. Exploration of indirect costs due to reduced economic productivity would be needed to estimate the cost impact from a societal perspective. Finally, we have not approached the question of mechanism here. Although depression is associated with greater impacts of pain, essentially poorer health and functioning, and greater health care costs, we can only speculate about the processes that underlie these links. Our study was cross-sectional so cannot identify the order of events or causal relations between variables.

Elsewhere, we have proposed the application of an integrated theoretical model^23^ that combines core behavioural, cognitive, and emotional processes that likely underlie both common health problems, such as pain and depression, and ostensibly varied therapeutic approaches. Perhaps, this kind of approach, which sees pain and depression as rooted in the same psychological and biological processes, rather than as separate disorders that co-occur, could help untangle the problems examined here. Such processes might include general psychological avoidance or what is known as “experiential avoidance” and failures in goal-directed engagement, both components of what is referred to as psychological inflexibility.^23^ A clue to the relevance of this model is here in the data showing an association between depression and acceptance of pain, the opposite process to experiential avoidance.

The findings of this study indicate a need for action. Further research is required to fill gaps in our understanding of the links between chronic pain and depression and the ways in which they combine to impact health and health care use. Such research may in turn contribute to treatment and health service developments. There is evidence that psychological treatments for chronic pain can reduce depression,^19,39^ in addition to improving physical functioning. Such treatment could be further developed and made more available. Of course, the other option is to develop treatment specifically targeted on depression in this population. It is not clear which, a depression-specific approach or a broader psychological approach focused on shared processes underlying both problems, will produce the more effective and cost-effective result.

## Conflict of interest statement

The authors have no conflicts of interest to declare.
